# Supplementary material for: Structural basis for substrate recognition and cleavage by the dimerization-dependent CRISPR–Cas12f nuclease
Source: Nucleic Acids Res. 2021 Mar 25;49(7):4120–8. doi: 10.1093/nar/gkab179 (PMC8053092; doi:10.1093/nar/gkab179)
Supplement: gkab179_Supplemental_Files [file gkab179_supplemental_files.zip › Supplementary_Materials_v4.pdf]

Supplementary Materials

SUPPLEMENTARY FIGURES

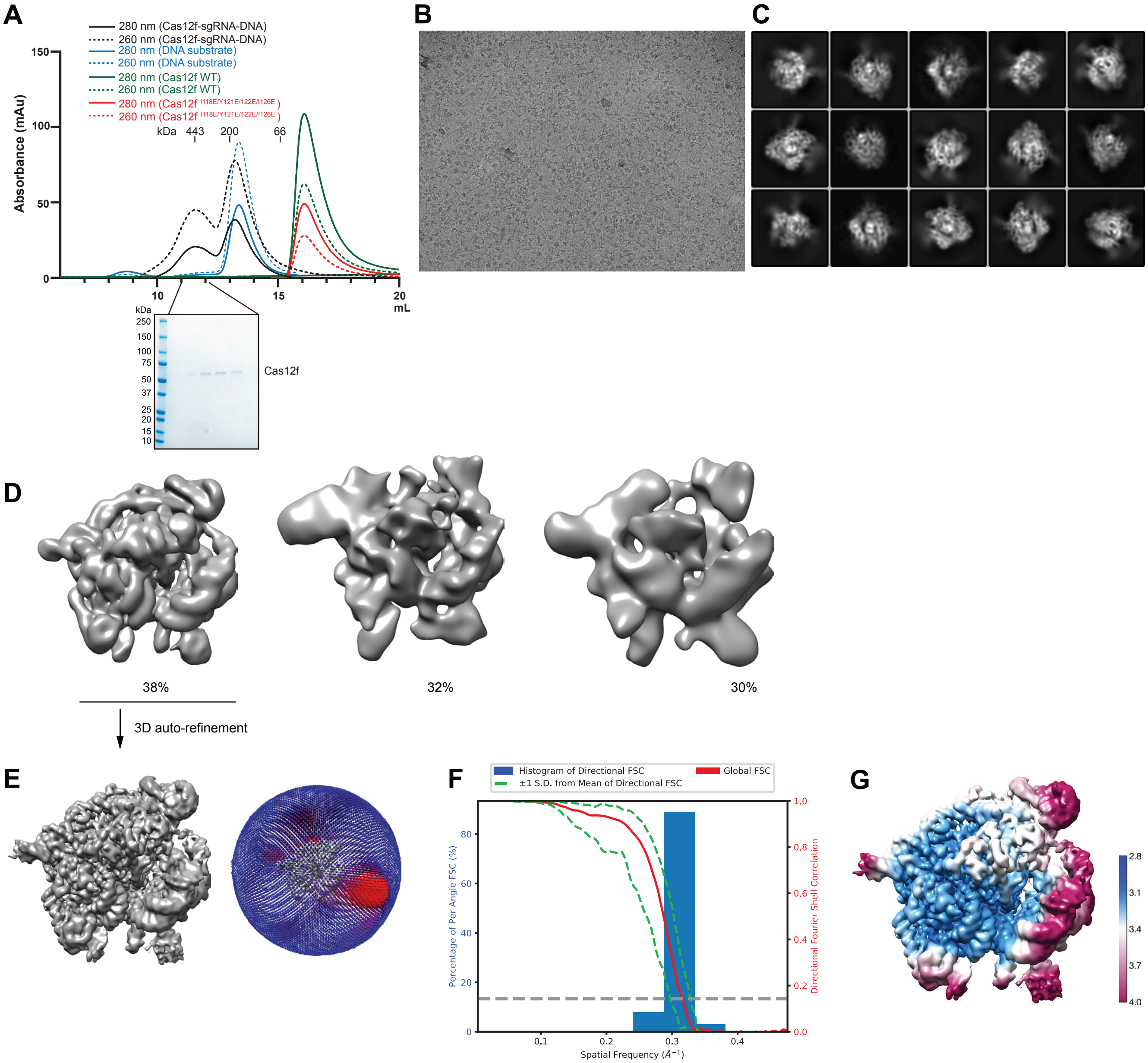

Figure S1. Sample preparation and cryo-EM of the Cas12f-sgRNA-target DNA ternary complex. **(A)** Superimposed size exclusion chromatography profiles of the Cas12f-sgRNA-target DNA ternary complex (black), DNA substrate only (blue), wild type Cas12f (green) and a Cas12f dimer interface mutation (I118E/Y121E/Y122E/Y126E) (red), with UV absorbance curves at 280 nm and 260 nm shown in solid and dashed lines, respectively. Peak positions and the molecular weights of standard marker proteins are indicated. Shown below is an SDS-PAGE analysis of elution fractions corresponding to the Cas12f-sgRNA-target DNA ternary complex. **(B)** A representative raw cryo-EM micrograph of the Cas12f-sgRNA-target DNA complex from a total of 2450 micrographs. **(C)**

Representative 2D class averages from a total of 100 images. **(D)** Three major classes from 3D classification. **(E)** 3D refinement for Class I with angular distribution of particles. **(F)** Plot of the global half-map FSC (solid red line) and spread of directional resolution values (±1σ from mean, green dotted lines; the blue bars indicate a histogram of 100 such values evenly sampled over the 3D FSC). FSC plot for the reconstruction suggests an average resolution of 3.1 Å. **(G)** Local resolution map for the reconstruction in **E**.

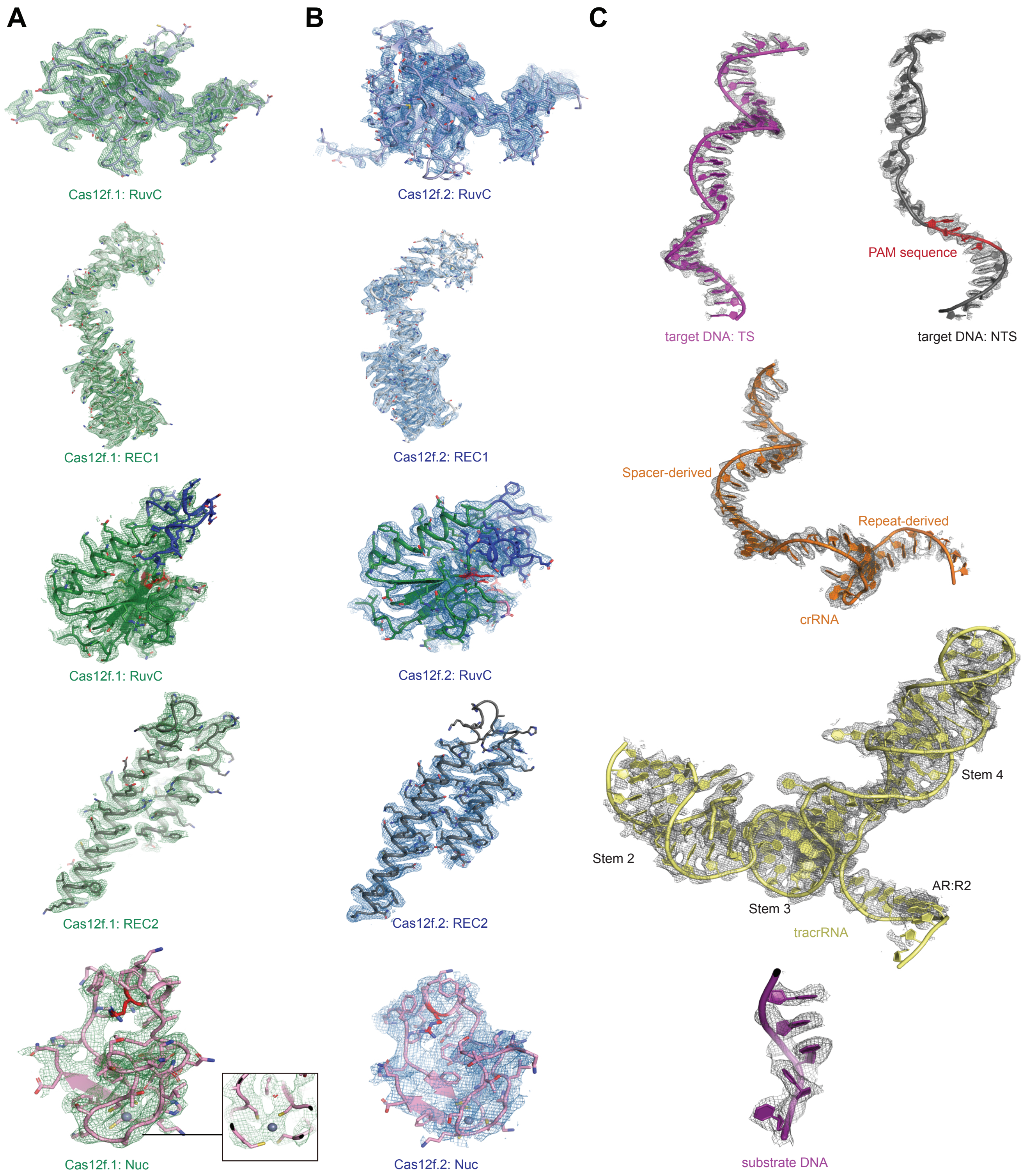

Figure S2. Detailed cryo-EM density map of the Cas12f-sgRNA-target DNA complex with atomic model fitted in. Cryo-EM density map (in mesh) of each domain in Cas12f.1 **(A)** and Cas12f.2 **(B)**, and each chain of nucleic acids **(C)** with atomic model fitted in.

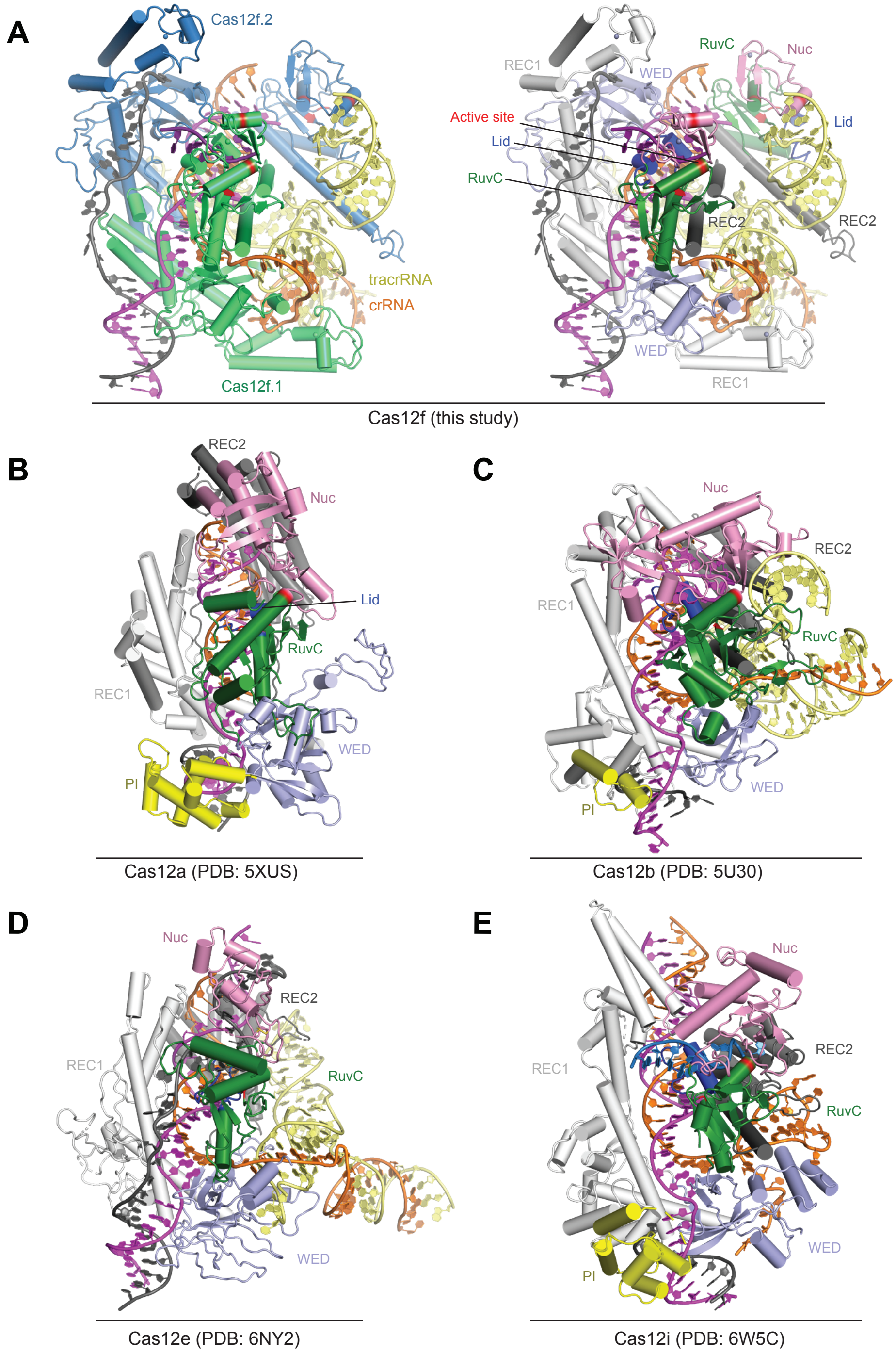

Figure S3. Structural comparison between Cas12f and other Cas12 nucleases. (A) Structure of Cas12f-sgRNA-target DNA complex. Left: two monomers of Cas12f are colored in green and blue, respectively. Right: each domain of Cas12f is color-coded as labeled. **(B-E)** Structure of Cas12a-crRNA-target DNA **(B)**, Cas12b-crRNA-target DNA **(C)**, Cas12e-crRNA-target DNA **(D)**, and Cas12i-crRNA-target DNA **(E)** with each domain color-coded as labeled.

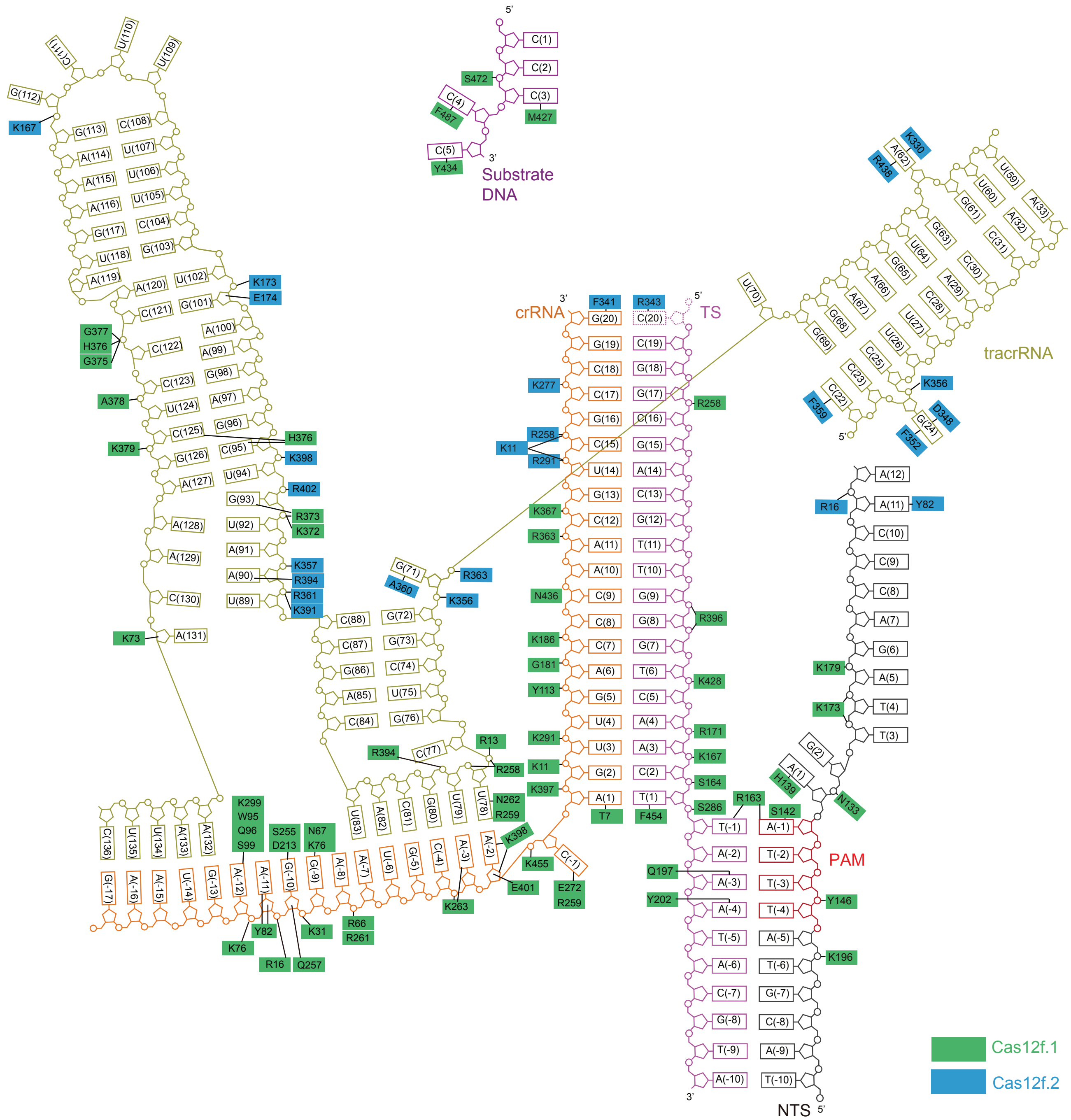

Figure S4. Schematic of the sgRNA and target DNA and their interactions with Cas12f monomers. Residues with green background are from Cas12f.1, while with blue background are from Cas12f.2. Interactions are indicated by black lines.

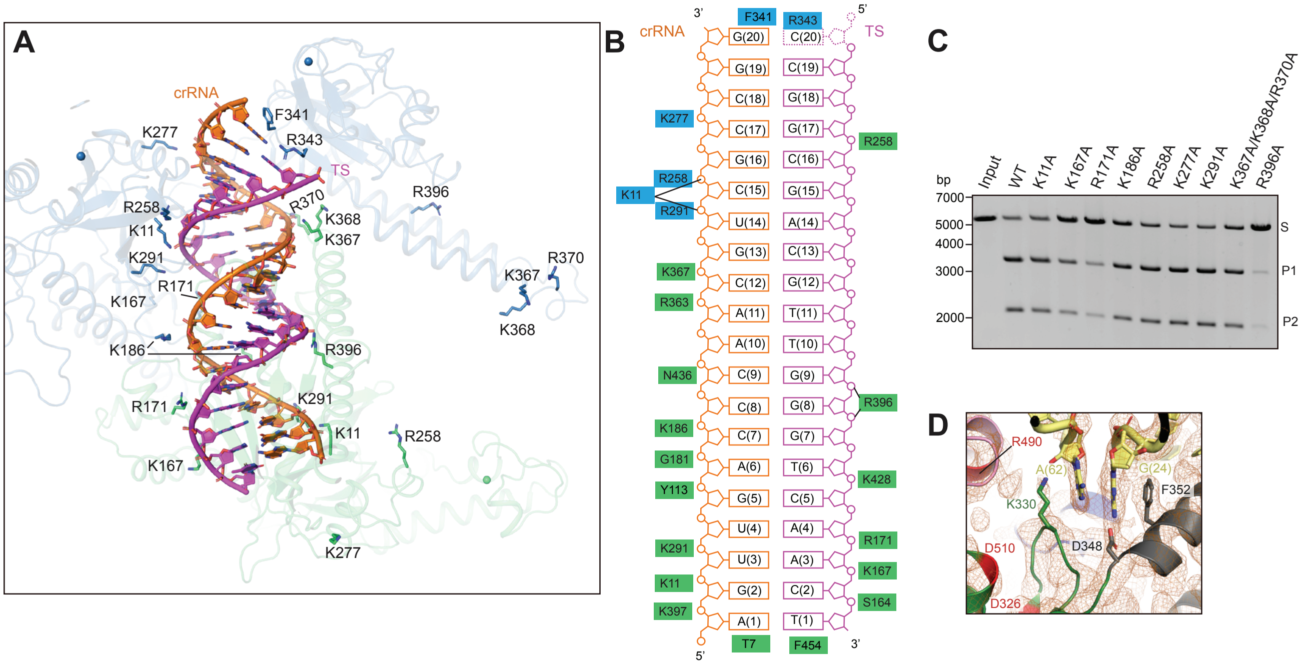

Figure S5. Interactions between Cas12f proteins and nucleic acids in the Cas12f-sgRNA-target DNA complex. (A,B) Structure **(A)** and schematic **(B)** showing the interactions between Cas12f and the crRNA-target DNA heteroduplex. **(C)** Substrate DNA cleavage assay using wild-type Cas12f and Cas12f with mutations in the positively charged residues which are involved in the recognition of the crRNA-target DNA heteroduplex. The results shown are representative of three experiments. **(D)** Interaction between tracrRNA and the RuvC active site of Cas12f.2.

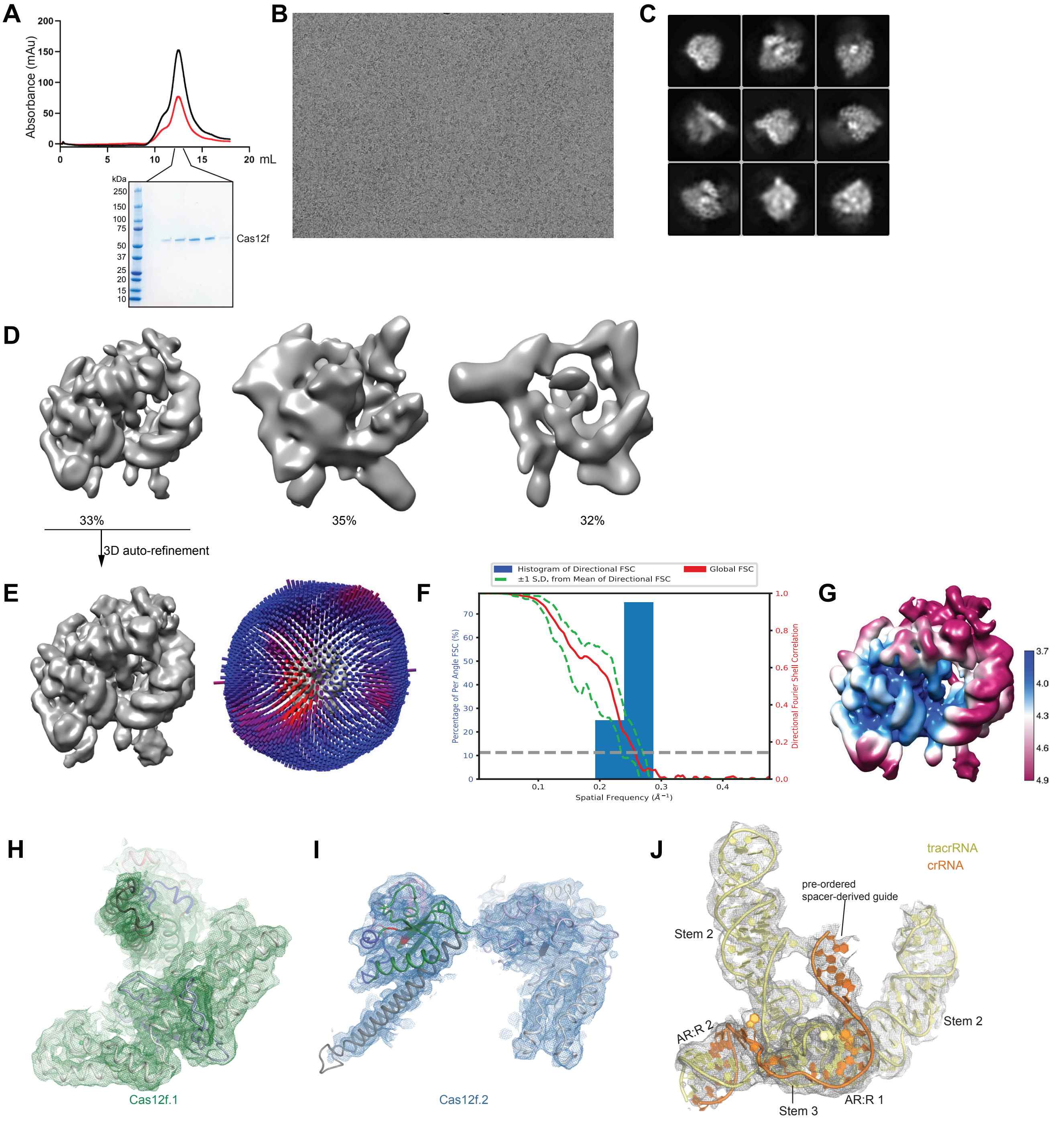

Figure S6. Sample preparation and cryo-EM of the Cas12f-sgRNA binary complex. (A) Purification of the Cas12f-sgRNA complex by size exclusion chromatography (SEC). UV absorbance curves at 280 nm and 260 nm are shown in red and black, respectively. SDS-PAGE analysis is shown below the SEC profile. **(B)** A representative raw cryo-EM micrograph of the Cas12f-sgRNA complex from a total of 1391 micrographs. **(C)** Representative 2D class averages from a total of 100 images. **(D)** Three major classes from 3D classification. **(E)** 3D refinement for Class I with angular distribution of particles. **(F)** Plot of the global half-map FSC (solid red line), and spread of directional resolution values (±1σ from mean, green dotted lines; the blue bars indicate a histogram of 100 such values

evenly sampled over the 3D FSC). FSC plot for the reconstruction suggests an average resolution of 3.9 Å. **(G)** Local resolution map for the reconstruction in **E**. **(H-J)** Cryo-EM density map (in mesh) of Cas12f.1 **(H)**, Cas12f.2 **(I)**, and sgRNA **(J)** with atomic model fitted in.

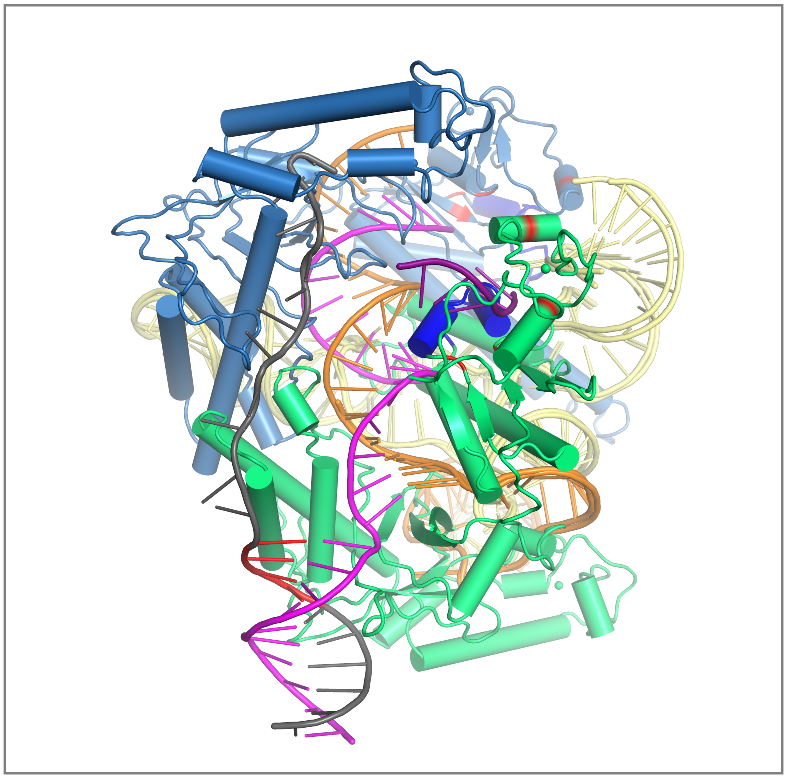

Movie S1. Conformational changes in Cas12f-sgRNA upon target DNA binding. This movie shows structural morphing of Cas12f-sgRNA before and after target DNA binding, in the same view as shown in **Fig. 5B**.

Table S1. Oligonucleotides utilized in assembly Cas12f-sgRNA-target DNA complex

Oligonucleotides	Sequences
sgRNA	CUUCACUGAUAAAGUGGAGAACCGCUUCACCAAAAGCUGUCCCUUAGGGG AUUAGAACUUGAGUGAAGGUGGGCUGCUUGCAUCAGCCUAAUGUCGAGAA GUGCUUUCUUCGGAAAGUAACCCUCGAAACAAAUUCAUUUUUCCUCUCCAA UUCUGCACAAGAAAGUUGCAGAACCCGAAUAGACGAAUGAAGGAAUGCAAC AGUUGACCCAACGUCGCCGG
Target strand	GCTGATGCATCTAGATTGTGCACGCCGGCGACGTTGGGTCAACTTAAATACG TAAGGTGC
Non-target strand	GCACCTTACGTATTTAAGTTGACCCAACGTCGCCGGCGTGCACAATCTAGAT GCATCAGC

Note: For sgRNA, the tracrRNA, linker, and crRNA regions are indicated in black, grey and orange, respectively.
